# Transcriptome analysis of the 2,4-dichlorophenoxyacetic acid (2,4-D)-tolerant cotton chromosome substitution line CS-B15sh and its susceptible parental lines *G. hirsutum* L. cv. Texas Marker-1 and *G. barbadense* L. cv. Pima 379

**DOI:** 10.3389/fpls.2022.910369

**Published:** 2022-08-22

**Authors:** Loida M. Perez, Ramil Mauleon, Mark A. Arick, Zenaida V. Magbanua, Daniel G. Peterson, Jeffrey F. D. Dean, Te Ming Tseng

**Affiliations:** ^1^Department of Biochemistry, Molecular Biology, Entomology & Plant Pathology, Mississippi State University, Starkville, MS, United States; ^2^Faculty of Science and Engineering, Southern Cross University, East Lismore, NSW, Australia; ^3^Institute for Genomics, Biocomputing and Biotechnology, Mississippi State University, Starkville, MS, United States; ^4^Department of Plant and Soil Sciences, Mississippi State University, Starkville, MS, United States

**Keywords:** herbicide resistance mechanism, plant physiology, abiotic stress tolerance, transcriptome analysis, RNA sequencing, auxin response and signaling, herbicide metabolism, Illumina sequencing

## Abstract

The cotton chromosome substitution line, CS-B15sh, exhibits 41% lower injury from 2,4-D when applied at the field recommended rate of 1.12 kg ae ha^−1^ (1×) than does Texas Marker-1 (TM-1). CS-B15sh was developed in the genetic background of *Gossypium hirsutum* L. cv TM-1 and has chromosome introgression on the short arm of chromosome 15 from *Gossypium barbadense* L. cv. Pima 379. In a previous experiment, we observed reduced translocation of [^14^C]2,4-D outside the treated leaf tissue in CS-B15sh, which contrasted with an increased translocation of the herbicide in the tissues above and below the treated leaf in TM-1. Our results indicate a potential 2,4-D tolerance mechanism in CS-B15sh involving altered movement of 2,4-D. Here, we used RNA sequencing (RNA-seq) to determine the differential expression of genes between 2,4-D-challenged and control plants of the tolerant (CS-B15sh) and susceptible lines (TM-1 and Pima 379). Several components of the 2,4-D/auxin-response pathway—including ubiquitin E3 ligase, PB1|AUX/IAA, ARF transcription factors, and F-box proteins of the SCF^TIR1/AFB^ complex—were upregulated with at least threefold higher expression in TM-1 compared with CS-B15sh, while both Pima 379 and TM-1 showed the same fold change expression for PB1|AUX/IAA mRNA. Some genes associated with herbicide metabolism, including flavin monooxygenase (Gohir.A01G174100) and FAD-linked oxidase (Gohir.D06G002600), exhibited at least a twofold increase in CS-B15sh than in TM-1 (the gene was not expressed in Pima 379), suggesting a potential relationship between the gene’s expression and 2,4-D tolerance. It is interesting to note that glutathione S-transferase was differentially expressed in both CS-B15sh and Pima 379 but not in TM-1, while cytochrome P450 and other genes involved in the oxidation–reduction process were significantly expressed only in CS-B15sh in response to 2,4-D. Gene set enrichment analysis on the union DEGs of the three cotton genotypes revealed the depletion of transcripts involved in photosynthesis and enrichment of transcripts involved in ABA response and signaling.

## Introduction

*Gossypium hirsutum* and *Gossypium barbadense* are two cultivated species of allotetraploid cotton. While *G. hirsutum* accounts for more than 90% of Upland cotton production worldwide, *G. barbadense* is superior in fiber quality, producing extra-long fibers for superior textile products (Saha et al., 2006; Li et al., 2015; Liu et al., 2015). *G. hirsutum* L. is known to be sensitive to 2,4-dichlorophenoxyacetic acid (2,4-D), and understanding the genetics of herbicide tolerance and identification of specific gene(s) involved at the molecular level is crucial for the development and genetic improvement of herbicide-resistant commercial cotton. A chromosome substitution line CS-B15sh was developed in the genetic background of Texas Marker-1 or TM-1 (*G. hirsutum* L.) with introgressions on the short arm of chromosome 15 from *G. barbadense* L. cv. Pima 379 via hybridization, cytogenetic analysis of progeny, and molecular marker selection (Stelly et al., 2005; Saha et al., 2012). In previous experiments, CS-B15sh exhibited reduced herbicide injury compared to TM-1 cotton seedlings when treated with 2,4-D at 1× field rate in greenhouse and field conditions (Perez, 2021). With the complex allotetraploid genomes of the two *Gossypium* species in hand and recombination and segregation data for genomic regions during the development of chromosome substitution lines, complementation of alleles leading to the identification of good recombinants can be a source of a novel gene for cotton genetic improvement on traits like herbicide tolerance (Saha et al., 2006; Li et al., 2015; Zhang et al., 2015).

2,4-D is a popular synthetic auxin that kills unwanted dicot plants (Schulz and Sehobye, 2016). At very low concentrations, 2,4-D mimics natural auxin in promoting cell division and elongation, while it exhibits herbicidal activity at high concentrations (Grossmann, 2000; Song, 2014). High doses of 2,4-D applied to sensitive dicots result in abnormal growth, premature senescence, and tissue decay (Grossmann, 2010). The herbicide is believed to act at multiple sites once the compound is absorbed by the plant (Gunsolus and Curran, 1999). It was reported that the mechanism of action of 2,4-D involves the activation of the auxin receptor system resulting in upregulation of auxin responses in the plant, increased ethylene production, and upregulation of ABA biosynthesis (Song, 2014; Schulz and Sehobye, 2016). High doses of auxin have been shown to result in chloroplast damage and progressive chlorosis leading to membrane leakage, overproduction of reactive oxygen species (ROS), localized necrosis, and cell death (Grossmann, 2000; Schulz and Sehobye, 2016).

At the molecular level, tolerance to 2,4-D in wild radish has been associated with mutations in ABCB-type auxin transporters in the plasma membrane; the mutated transporters reduced the herbicide translocation rate in wild radish (Goggin et al., 2016). The biosynthesis and signaling by natural auxin, IAA, which promotes normal cell division, elongation, and normal plant growth, is tightly regulated in plants. At low auxin concentrations, Aux/IAA proteins bind auxin-response factors (ARFs), preventing the transcription and expression of auxin-inducible genes (Song, 2014). However, 2,4-D, when present at high concentrations, acts as molecular glue that binds Aux/IAA to the F-box transport inhibitor response 1 (TIR1) protein and mediates proteasome degradation of Aux/IAA proteins (Dharmasiri et al., 2005). The TIR1 protein appears to have a critical role in auxin (2,4-D) signaling (Parry et al., 2009). This leaves free ARF proteins that bind to auxin-response elements (AuxRes) and facilitates transcription of auxin-response genes leading to herbicidal responses (Song, 2014). Interestingly, TIR1 associated with ubiquitin-mediated auxin signaling was downregulated at low 2,4-D concentrations in Arabidopsis (Raghavan et al., 2006). In addition, the transcriptional gene silencing pathway was affected in response to 2,4-D herbicide treatment, which led to increased susceptibility to the herbicide in Arabidopsis (Markus et al., 2021). Other transcriptional responses as a result of herbicide treatment in other species have been associated with nutrient limitation due to the increased expression of genes involved in alternative carbon and nitrogen source metabolism (Teixeira et al., 2006). The herbicidal effects of 2,4-D on ABA biosynthesis and signaling have been reported in Arabidopsis (Raghavan et al., 2005, 2006). In citrus, ABA levels detected by high-performance liquid chromatography–tandem mass spectrometry (HPLC-MS/MS) were significantly increased with post-harvest 2,4-D treatment (Ma et al., 2014). In salt-tolerant rice cultivar, 2,4-D treatment stimulated the synthesis of the stress hormone ABA regulating specific antiporter activities in the cell associated with the efflux and influx of ions in the plasma membrane (Islam et al., 2017). The effects of 2,4-D treatment on the reduction of photosynthetic process have been reported as early as 1950s (Wedding et al., 1953; Williams and Dunn, 1966). In photosynthetic cyanobacterium *Nostoc muscorum* Meg 1, transcription levels of several photosynthesis-related genes were compromised with the increasing dose of 2,4-D applied (Sachu et al., 2022). In plants, a significantly reduced photosynthesis and stimulated ABA levels were detected in the weed species *Erigeron canadensis* following 2,4-D herbicidal application (McCauley et al., 2020). The upregulation of NCED, key enzyme in ABA biosynthesis, is said to be the principal step in the synthetic auxin herbicide mode of action (McCauley et al., 2020).

However, their findings also indicate that the increase in ABA levels were independent of the increase in ethylene which is contrary to previous reports on the pathways leading to auxin herbicidal response and symptoms in plants (Grossmann, 2000; Hansen and Grossmann, 2000). However, the general downregulation of transcript abundance of photosynthesis-related genes is proposed to be the result of ABA accumulation which then leads to loss of photosynthetic capacity, deregulation of growth, and plant death (Gaines, 2020).

Several mechanisms have been described for herbicide tolerance in weeds and cultivated crops, including upregulation or downregulation of families of enzymes, such as cytochrome P450s, glutathione *S*-transferases (GSTs), and glycosyltransferases; all of these families are involved in herbicide degradation and metabolism of 2,4-D into non-toxic forms (Ohkawa et al., 1999; Werck-Reichhart et al., 2000; Busi and Powles, 2017). Herbicide tolerance to 2,4-D in the weed species *Raphanus raphanistrum* was shown to be conferred by a single dominant locus that depicted a nuclear inheritance pattern (Busi and Powles, 2017). Similarly, a single codominant gene was responsible for 2-4-D tolerance in prickly lettuce (*Lactuca serriola* L.) (Riar et al., 2011). Reduced translocation and enhanced metabolism were observed in 2,4-D-resistant corn poppy (*Papaver rhoeas*) populations in Spain (Torra et al., 2017). Rapid metabolism of 2,4-D was also observed in common waterhemp (*Amaranthus tuberculatus*), showing tolerance to the herbicide (Shyam et al., 2019).

In cotton, 2,4-D tolerance was introduced by *Agrobacterium*-mediated transfer of a 2,4-D monooxygenase gene, *tfdA*, from *Alcaligenes eutrophus*, which degrades the active compound 2,4-D into non-toxic 2,4-dichlorophenol (Bayley et al., 1992). Dow AgroSciences released the first commercial 2,4-D-tolerant cotton varieties employing the Enlist technology which uses a transgene from bacteria encoding an aryloxyalkanoate dioxygenase that efficiently degrades 2,4-D to non-toxic derivatives (Wright et al., 2010). This technology has gained widespread acceptance and adoption by cotton farmers needing a new control technology for weeds that have developed tolerance to glyphosate while maintaining high seed and lint yield. Deployment of Enlist cotton has led to problems in areas where non-Enlist cotton is also grown. Non-Enlist cotton is highly sensitive to 2,4-D, and off-target spray drift injury incidents are increasing with concomitant damage claims (Byrd et al., 2016). To address this problem, alternative sources of 2,4-D tolerance that could be introduced into cultivated cotton without genetic engineering would be highly desirable.

High-throughput sequencing has enabled the rapid and efficient analysis of complex traits, including the genetic basis of tolerance mechanisms and the metabolic pathways that respond to the application of auxinic herbicides, like 2,4-D. Using this approach, specific-resistant allele variants of a cytochrome P450 have been associated recently with non-target site tolerance to 2,4-D in *A. tuberculatus* (Giacomini et al., 2020). In wild radish (*R. raphanistrum*), the RNA-seq analysis of herbicide-resistant plants treated with 500 g ae ha^−1^ 2,4-D amine revealed that the tolerance mechanisms included complex and population-specific changes in auxin signaling and elevated plant defense responses (Goggin et al., 2018). RNA-seq analysis has also been used to dissect glufosinate tolerance in *Amaranthus palmeri* and investigate global transcriptional changes associated with tolerance to the herbicide (Salas-Perez et al., 2018).

This study aims to understand the interaction of cotton with 2,4-D herbicide at the molecular level. We expect to improve our understanding of the genetics and molecular mechanisms of 2,4-D tolerance by inference from the analysis of specific DEGs that were upregulated or downregulated in the 2,4-D-tolerant line, CS-B15sh, compared to the 2,4-D-sensitive parental lines, TM-1 and Pima 379. We anticipate that this information will inform future breeding efforts to improve the tolerance of non-engineered Upland cotton to herbicide spray drift from adjoining agricultural fields.

## Materials and methods

### Plant materials and 2,4-D treatment

The chromosome substitution line CS-B15sh (31-4), *G. hirsutum* L. cv. TM-1, and *G. barbadense* L. cv. Pima 379 were used in the study. Seeds were obtained from the USDA−ARS Crop Science Research Laboratory, Mississippi State, MS, United States. Seedlings were established by sowing cotton seeds in 18-cell Landmark plastic trays (53 cm × 26 cm) (Landmark Plastic, Akron, OH, United States) containing soil (BX PROMIX Growing Medium 10280, BWI Companies, Inc., Nash, TX, United States) amended with 1–2 g of the basal fertilizer Osmocote Plus (The Scotts Company, Marysville, OH, United States). Seedlings (one per cell) were maintained in the greenhouse and watered once a day prior to treatment. The cotton seedlings were treated when plants reached the 4–5 leaf growth stage by spraying 2,4-D (Weedar 64, Nufarm Americas Inc., Alsip, IL, United States) at a rate of 1 lb. acre^−1^ (1.12 kg ae ha^−1^) in a controlled spray chamber equipped with a TP8002VS Even Flat Spray Tip (TeeJet^®^, Spraying System Co., Wheaton, IL, United States) calibrated to deliver 20 gallons acre^−1^ (GPA) at 40 psi. After herbicide application, the treated plants were allowed to dry for 1–2 h before being transferred back to the greenhouse; the drying period was included to prevent the transfer of volatilized herbicide to control plants (water-sprayed only). Irrigation was resumed on the treated seedlings after 24 h. The non-treated controls (water-sprayed) were planted and grown at the same time as the treated plants and were maintained in the same manner except for the 2,4-D treatment. The experiment was conducted following a completely randomized design with two treatments (2,4-D-treated and non-treated control) and four replications per treatment for each line (TM-1, Pima 379, and CS-B15sh). Herbicide injury was evaluated 21 days after herbicide application using the 0–100% injury scale by Behrens and Lueschen (1979). Leaf tissues (2nd true leaf) were collected from the treated and non-treated cotton seedlings 12 h after treatment (12 HAT) and were immediately frozen in liquid nitrogen and stored at −80°C prior to extraction of total RNA.

### RNA extraction, library preparation, and Illumina sequencing

Total RNA was extracted from the collected leaf tissues using a modified hot borate method (Wan and Wilkins, 1994). Double- and single-stranded DNA were removed from the RNA samples using deoxyribonuclease I (RQ1 RNase-Free DNase, 1000u, Promega Corporation, Madison, WI, United States). The RNA samples were further purified using the Qiagen RNeasy^®^ Mini Kit (Catalog No. 74104, Qiagen, Germantown, MD, United States). The quality of the total RNA was assessed by agarose gel electrophoresis, and the concentration was determined using a Nanodrop spectrophotometer (NanoDrop*^C^*, Thermo Scientific, Waltham, MA, United States).

The RNA samples then were used to construct cDNA libraries using the NEBNext^®^ Ultra II Directional RNA Library Prep Kit for Illumina with Sample Purification Beads (New England Biolabs, Ipswich, MA, United States) following the manufacturer’s instructions. The concentration and quality of cDNA libraries were determined using fluorometry (Qubit™ fluorometer, Invitrogen™ by Thermo Fisher Scientific, Carlsbad, CA, United States) and capillary zone electrophoresis (Agilent 2100 Bioanalyzer, Santa Clara, CA, United States), respectively. The manufacturer’s procedure described in the Agilent DNA 1000 Reagent kit (Agilent Technologies, Santa Clara, CA, United States) was used in checking the quality of cDNA libraries. The 24 RNA-Seq libraries (treated vs. control, three genotypes, four replicates), each with unique barcodes, were pooled and sequenced on three lanes (eight libraries per lane) of an Illumina HiSeq X-Ten (paired-end, 150 bp) (Illumina, San Diego, CA, United States).

### Identification and functional analysis of differentially expressed genes

Alignment of the raw reads to the reference *G. hirsutum* (AD1) TM-1 genome UTX_v2.1 (Chen et al., 2020) and quantification of transcript abundance were performed using Salmon v1.3.0 (Patro et al., 2017). The Salmon aligner which uses a couple forms of Bayesian inference was employed to estimate abundance with the ambiguously/multiple mapped reads (Hu et al., 2021). The R v4.0.2 package tximport (version 1.16.1) was used to convert the transcript-level data into gene-level analysis using the recommended expression normalization method (Soneson et al., 2016; R Core Team, 2020). Genes with low expression levels (average log-transformed counts per million less than 1) were filtered from further analysis (Bourgon et al., 2010). Differential gene expression analysis was conducted using the quasi-likelihood generalized log-linear model available from edgeR (version 3.30.3), a Bioconductor software package^1^ (Robinson et al., 2010). The response to 2,4-D was tested by pairwise comparison between the treated and non-treated controls of each line (CS-B15sh, TM-1, and Pima 379). Likewise, the differences in the responses of the three genotypes to 2,4-D were tested by pairwise comparison of the responses of each line to one another. The changes in basal gene expression were also tested by pairwise comparison of the non-treated controls of each line with one another. In a similar manner, changes in gene expression among the treated samples of each line were also identified using pairwise comparison. Genes that were significantly (FDR ≤ 0.05) different in response to 2,4-D in the CS-B15sh line vs. the two susceptible lines, TM-1 and Pima 397, and were not significantly different between TM-1 and Pima 397 were considered genes of interest. A composite DEG list (FDR < 0.001) for herbicide-treated vs. non-treated samples in all three cotton lines (Supplementary Table 1) was used in further downstream analyses such as hierarchical clustering (distance metric Pearson’s correlation, average linkage clustering), metabolic pathway/reaction analysis using CottonGen Tools^2^, MapMan (version 3.5.1R2) analysis (Thimm et al., 2004), and GO enrichment analysis using FGSEA package (release 3.14) with default settings of 1,000 permutations and *P*-value cutoff *P* < 0.05 in R Bioconductor^3^. Pathway analysis using the gene expression data for 59 selected DEGs was carried out using Pathway Tools Omics Dashboard (Paley et al., 2017). Functional annotation of the DEGs was obtained from the TM-1 genome UTX_v2.1 (Chen et al., 2020).

## Results

**FIGURE 1:**
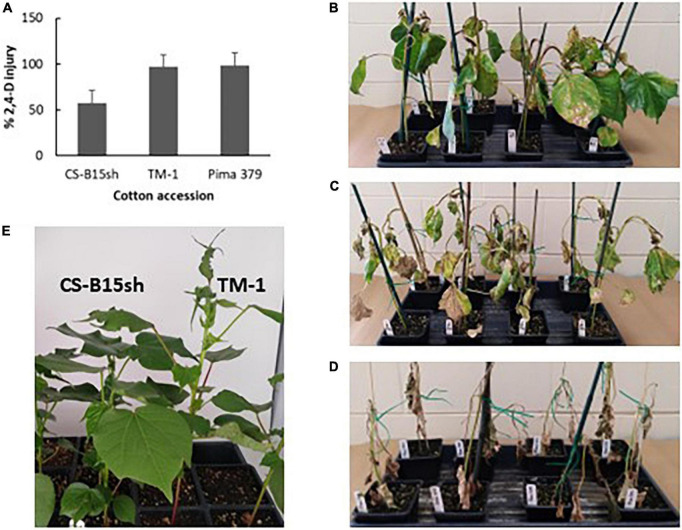
2,4-D herbicide injury and symptoms observed on CS-B15sh, TM-1, and Pima 379. Clockwise from upper left, **(A)** histogram of herbicide injury observed 21 days after application of 1× rate (1.12 kg ae ha^–1^) 2,4-D on 4–5 leaf stage cotton seedlings in the greenhouse; 2,4-D injury observed in CS-B15sh **(B)**, TM-1 **(C)**, and Pima 379 **(D)** after 2 weeks from herbicide spraying; **(E)** in another experiment, CS-B15sh and TM-1 sprayed with drift rate (0.05×) of 2,4-D herbicide at 5 weeks from spraying, with TM-1 showing leaf epinasty while CS-B15sh showing normal leaf growth.

This study determined that CS-B15sh plants treated with the recommended field rate (1.12 kg ae ha^−1^) of 2,4-D showed 58% herbicide injury on average, while TM-1 and Pima 379 plants exhibited 97 and 98.5% herbicide injury, respectively (Figure 1). Whereas CS-B15sh showed moderate leaf epinasty, stem curling, and necrosis 14 days after treatment, the TM-1 and Pima 379 plants showed severe injury with epinasty, leaf/stem curling, severe and widespread necrotic lesions, and plant death in some seedlings. Non-treated seedlings of all genotypes showed none of the visible injuries associated with 2,4-D treatment (data not shown).

### RNA sequencing and differential expression

**FIGURE 2:**
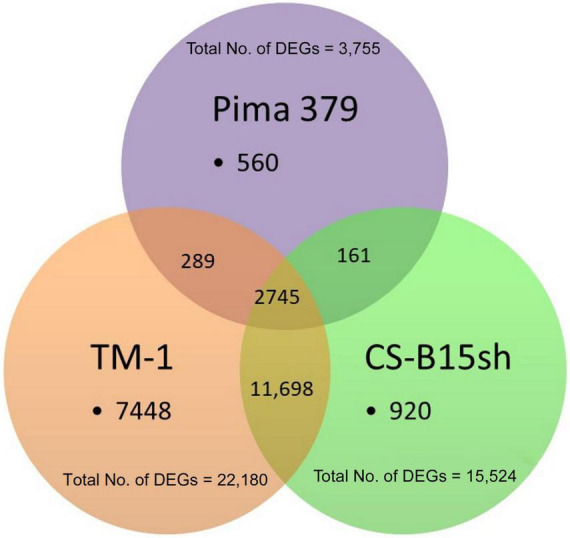
Distribution of differentially expressed genes (DEGs) (*N* = 23,821) with FDR *P*-value < 0.001 either in CS-B15sh, TM-1, or Pima 379 cotton lines in response to 2,4-D herbicide applied at 4–5 leaf stage cotton seedlings in the greenhouse.

Sequencing of the short-read Illumina libraries generated approximately 60 million fragments (average) per library. Analysis of the cDNA fragment libraries by capillary zone electrophoresis (Bioanalyzer) showed the expected 250–300 bp size range. The fragment sequences mapped to the *G. hirsutum* (AD1) TM-1 genome UTX_v2.1 at a rate of 61–74% with 43.5 million fragments per library aligned to the reference genome for cotton. A total of 23,821 genes were found in the samples, of which 920 (3.9%), 7,448 (31.3%), and 560 (2.4%) were exclusively expressed (herbicide-treated vs. untreated) in CS-B15sh, TM-1, and Pima 379, respectively (Figure 2). Common DEGs to both CS-B15sh and TM-1 were 11,698 (49.1%), while 161 (0.7%) were shared between CS-B15sh and Pima 379. A total of 289 (1.2%) DEGs were shared between TM-1 and Pima 379 only, while 2,745 (11.5%) exhibited significant differential expression in the three cotton lines used in the study. The total number of DEGs in response to 2,4-D treatment was 15,524, 22,180, and 3,755 in CS-B15sh, TM-1, and Pima 379, respectively.

The gene set enrichment analysis (2,745 DEGs, intersect) revealed the significant Gene Ontology (GO) terms involved in cotton’s response to 2,4-D, including regulation of transcription, catalytic activity, response to auxin, transferase activity, extracellular region, cellular amino acid metabolic process, plant-type cell wall organization, ion channel inhibitor activity, biosynthetic process, response to oxidative stress, and xenobiotic transmembrane transporter activity (Supplementary Table 2).

### The auxin herbicide response and signaling in cotton

**FIGURE 3:**
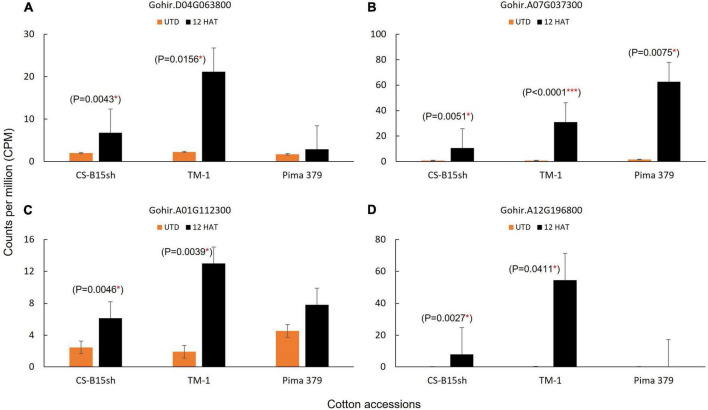
Number of transcripts detected in DEGs associated with the auxin (2,4-D) herbicide response pathway in plants; **(A)** Gohir.D04G063800, E3 ubiquitin-protein ligase; **(B)** Gohir.A07G037300, PB1 domain|AUX/IAA; **(C)** Gohir.A01G112300, auxin-response factor; and **(D)** Gohir.A12G196800; F-box domain. A number of transcripts for Gohir.D04G063800, Gohir.A01G112300, and Gohir.A12G196800 detected in Pima 379 are not significantly different. Analysis of variance and comparison of means based on Tukey–Kramer (HSD) was applied using JMP 14 statistical package (SAS Institute, NC, United States).

The molecular pathways for auxin and 2,4-D response in plants have been reported (Badescu and Napier, 2006; Hao and Yang, 2010; Song, 2014). Of the key molecular factors identified, no information is available in cotton, and knowing as well as understanding the several components involved is important in the genetic manipulation for herbicide response in Upland cotton. Since 2,4-D mimics the natural auxin hormone in plants, we found several genes reported significantly expressed and upregulated in the cotton lines treated with 2,4-D. This includes ubiquitin E3 ligase, F-box domain, PB1 domain|AUX/IAA proteins, and auxin-response factor (Table 1). The ubiquitin E3 ligase genes (RBR family and RNF126-like protein) exhibited twofold higher in TM-1 compared to CS-B15sh. For instance, Gohir.A05G142700 showed a 106-fold change in TM-1 while CS-B15sh showed 53.3. Similarly, Gohir.D04G063800 showed 9.2 in TM-1, while 3.4-fold change was observed in the 2,4-D-treated CS-B15sh plants. Both genes were not significant in Pima 379. The PB1|AUX/IAA domains, Gohir.A08G062000, and Gohir.A07G037300 exhibited similar fold change in both TM-1 and Pima 379, and the level of expression is threefold higher (FC, 40–49) compared with CS-B15sh (FC, 13–16). Gohir.D08G261100 was not significant in Pima 379 but exhibited threefold higher expression in TM-1 (FC, 72) than CS-B15sh (FC, 23). The auxin-response factor, Gohir.A01G112300, was significant in TM-1 with a sevenfold change, while the gene was not significant in CS-B15sh and Pima 379. A gene coding for F-box domain (Gohir.A12G196800) is highly expressed in TM-1 with a 182-fold change, 3× higher than CS-B15sh (FC, 53). The number of transcripts in terms of counts per million (CPM) detected on each gene corresponded with the fold change of expression described above (Figure 3). E3 ubiquitin ligase showed 8× higher transcript level after 12 h in TM-1 while 2× higher in CS-B15sh compared to the baseline transcript level of untreated plants (Figure 3A). The PB1|AUX/IAA domain exhibited the same increase of transcript level (38×) at 12 h for TM-1 and Pima 379, while only a 12× increase was shown in CS-B15sh (Figure 3B). Interestingly, the F-box domain exhibited a sharp rise in transcript level with a 180-fold increase after 12 h of 2,4-D treatment in TM-1. The gene was not significant in Pima 379 and showed 38× higher expression in CS-B15sh (Figure 3D). On the contrary, the auxin-response factor transcript amounts were 6× higher at 12 h in TM-1, while the gene was not significant in CS-B15sh and Pima 379 (Figure 3C). Other DEGs known as hormone-responsive genes were also significantly upregulated, including AP2/ERF domain protein (Gohir.A13G053300) with a 65-fold change in CS-B15sh. At the same time, oxoglutarate/iron-dependent dioxygenase (Gohir.A13G155500) exhibited a 442-fold increase in expression in treated TM-1 seedlings, 7× higher than the transcript level detected in the treated CS-B15sh seedlings (Table 1).

**TABLE 1:** Differentially expressed genes of the 2,4-D auxin-response pathway and other hormone-responsive genes in the cotton lines, CS-B15sh, TM-1, and Pima 379 in response to 2,4-D^a^.

Gene ID	Annotation	CS-B15sh		TM-1		Pima 379	
		FC	FDR	FC	FDR	FC	FDR
Gohir.A05G142700	E3 ubiquitin ligase RBR family	53.27	1.01304E-06	105.57	2.75142E-08	3.41	0.055206147
Gohir.D04G063800	E3 ubiquitin-protein ligase RNF126-like	3.39	0.000648236	9.25	6.69535E-07	1.67	0.13905765
Gohir.A12G196800	F-box domain	46.18	1.05598E-05	182.22	1.00358E-07	2.68	0.309769358
Gohir.D08G261100	PB1 domain|AUX/IAA protein	22.70	1.65682E-06	72.07	2.52846E-08	55.58	1.3395E-06
Gohir.A08G062000	PB1 domain|AUX/IAA protein	15.99	1.18976E-07	48.63	1.862E-09	52.43	8.08049E-09
Gohir.A07G037300	PB1 domain|AUX/IAA protein	12.86	2.96185E-07	39.61	2.63972E-09	38.30	2.04864E-08
Gohir.A01G112300	Auxin response factor	2.52	0.002911941	6.77	1.33197E-06	1.72	0.076405509
Gohir.A13G053300	AP2/ERF domain	64.72	2.2273E-05	48.59	1.59398E-05	11.32	0.001791259
Gohir.A13G155500	Oxoglutarate/iron-dependent dioxygenase	62.99	0.001143269	422.12	2.76668E-07	3.50	0.298534768

^a^FC, fold change; FDR, false discovery rate adjusted *p*-value.

Levels of gene expression are detected in cotton lines CS-B15sh, TM-1, and Pima 379 at 12 h after spraying 1× (1.12 kg ae ha^−1^) 2,4-D in 4–5 leaf stage cotton seedlings in the greenhouse.

### Effects on photosynthesis and abscisic acid metabolism

**FIGURE 4:**
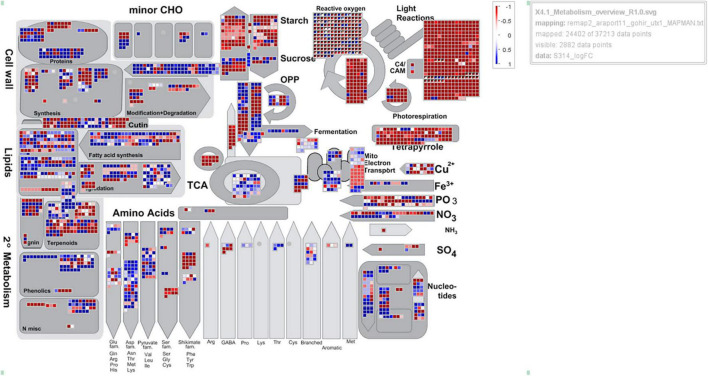
MapMan analysis showing the overview of metabolic pathways represented by genes differentially expressed (2,4-D-treated vs. untreated) in the cotton line CS-B15sh at 12 h after treatment with 1× rate of herbicide 2,4-D. Each data point (square) represents a DEG gene. Blue and red colors indicate the DEGs are upregulated and downregulated, respectively.

A total of 193 genes associated with photosynthesis were significant in all three cotton lines treated with 2,4-D (Supplementary Table 3). Of these, 95% were downregulated and composed of DEGs involved in photosystems I and II, light-harvesting complex, chlorophyll biosynthesis, ATP synthesis, and electron transport chain. In Pima 379, the top five significantly downregulated DEGs were involved in PS I light-harvesting complex, chlorophyll biosynthesis, and ATP synthesis, while TM-1 also included PS I light-harvesting and PS II DEGs. CS-B15sh also exhibited the most significant downregulation of PS I light-harvesting followed by DEGs for the electron transport chain. From the major group of downregulated genes, 2Fe-2S ferredoxin-type iron-sulfur binding domain (Gohir.A13G222600), which is involved in the electro-transport chain, is upregulated in all the herbicide-treated cotton lines. At the same time, Mog1/PsbP of PS II, an extrinsic membrane component, showed upregulation of gene expression in Pima 379 but was downregulated in both CS-B15sh and TM-1. The small group of DEGs for photosynthesis were composed of upregulated genes involved in electron transfer activities, including ATP synthase (Gohir.D01G067400 and Gohir.A01G080200), 2Fe-2S ferredoxin-type iron-sulfur binding domain (Gohir.D13G227400), and Ferredoxin–NADP reductase (Gohir.A05G110400), with two DEGs of unknown function from chromosome 19 (D05) of *G. hirsutum* L., Gohir.D05G110800 and Gohir.D05G0837000. Photosystem I reaction center subunit IX (PsaJ) superfamily is upregulated in both Pima 379 and TM-1 while exhibiting downregulation of gene expression in CS-B15sh. An overview of metabolic pathways and gene regulatory networks observed in the three cotton lines is presented in Figure 4 and Supplementary Figures 1, 2. Generally, almost all genes found and expressed in the cotton lines and involved in the Calvin cycle, photorespiration, PS I, and PS II exhibited negative log2 fold changes (red boxes) in the three genotypes. More downregulation of photosynthesis-related genes was observed in TM-1 compared to CS-B15sh and Pima 379 (Supplementary Figure 3).

The effects of 2,4-D herbicide on ABA signaling and biosynthesis in cotton showed 11 of 18 DEGs were upregulated, exhibiting positive log2 fold change and involving genes for abscisic acid binding and receptor activity (Supplementary Table 4). The top five highly expressed ABA-associated genes encoded mainly Bet v I/Major latex protein (START-like domain superfamily) and were significantly upregulated in both TM-1 and CS-B15sh, except for Gohir.A10G169466, which showed the highest 7.7 log2 fold change only found in TM-1. Aspartic peptidase A1 family (Gohir.A09G177900) was significantly upregulated (FDR < 0.001) in the three cotton lines evaluated with log2 fold change of 1.7, 2.1, and 1.6 for Pima 379, CS-B15sh, and TM-1, respectively (Supplementary Table 4). The rest of the ABA genes found were downregulated including nodulin-related protein 1, dual-specificity phosphatase, protein SHORT HYPOCOTYL IN WHITE LIGHT 1, aspartic peptidase A1 family, and forkhead-associated (FHA) domain. The patterns of gene expressions of DEGs associated with ABA metabolism and response are similar in the cotton lines.

### DEGs responsive to 2,4-D in CS-B15sh

**FIGURE 5:**
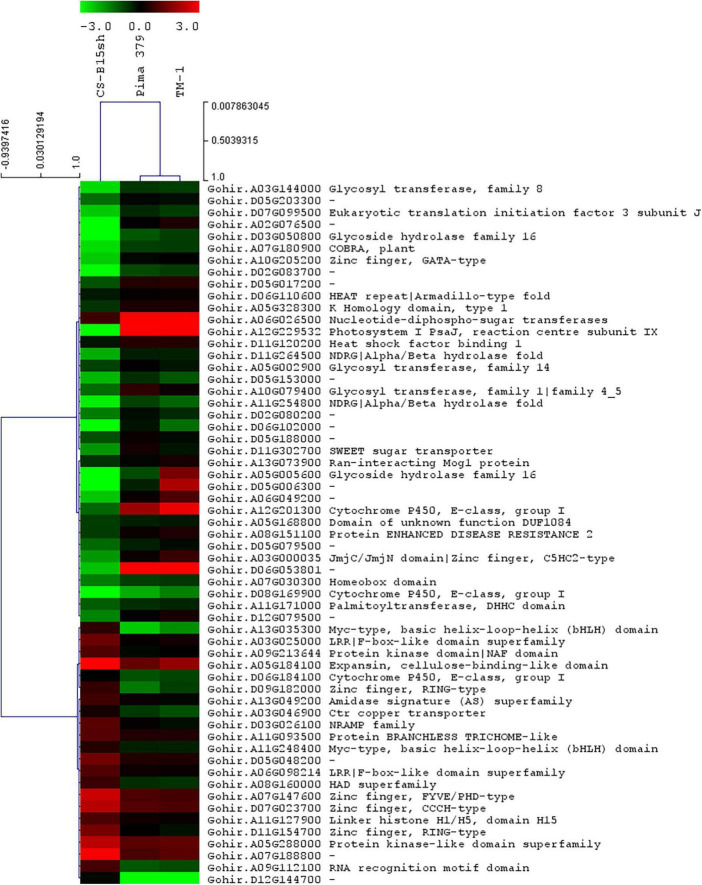
Gene co-expression heatmap with hierarchical clustering of 59 genes differentially expressed (12 HAT treated vs. untreated) in the three cotton lines, CS-B15sh, TM-1, and Pima 379, sprayed with 1× rate 2,4-D herbicide. DEGs with “–” indicate unknown function.

**FIGURE 6:**
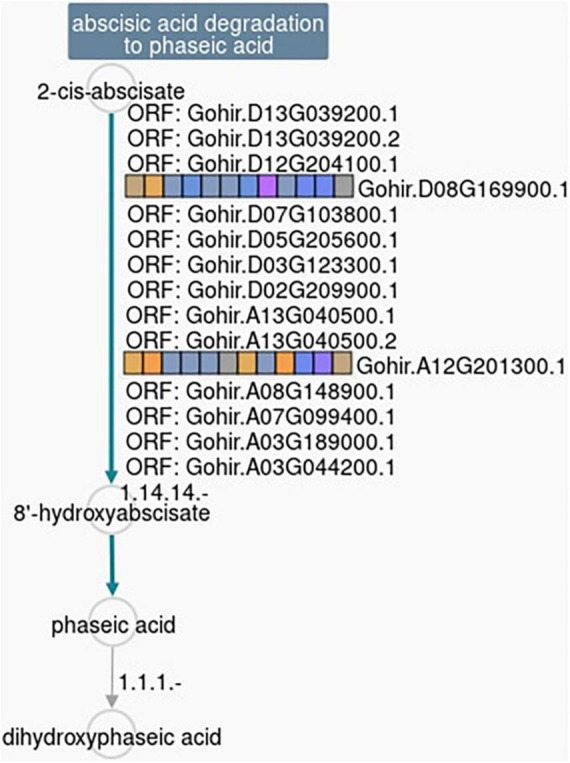
Ptools pathway analysis shows that two of the 59 DEGs responsive to 2,4-D treatment in the herbicide-tolerant cotton line CS-B15sh (Gohir.D08G169900 and Gohir.A12G201300) are involved in the 2-ci-abscisate → 8′-hydroxyabscisate conversion step in the abscisic acid degradation pathway. The pathway collage generated using the Pathway Tools Omics Dashboard (https://ptools.cottongen.org).

Based on the analysis of the different responses of each cotton line to 2,4-D treatment, 59 genes passed the response comparison filter with significantly different (FDR ≤ 0.05) responses to 2,4-D treatment in CS-B15sh compared to susceptible lines but not significantly different (FDR > 0.05) between the susceptible lines (Table 2). Functional annotation revealed that the genes were involved in protein and DNA binding, transmembrane transporter activity, transferase activity, hydrolase activity, photosystem 1, iron ion binding, and nucleosome and transcription processes. Fifty-one genes were significantly (FDR ≤ 0.05) differentially expressed in the CS-B15sh response to 2,4-D, with 18 upregulated and 33 downregulated (Table 2). The top three upregulated DEGs in the CS-B15sh response to 2,4-D are expansin, cellulose-binding-like domain (Gohir.A05G184100), zinc finger, FYVE/PHD-type (Gohir.A07G147600), and a novel cotton gene Gohir.A07G188800 of unknown function with log2 fold change of 4.4, 2.4, and 2.9, respectively. The top five downregulated genes in the CS-B115sh response to 2,4-D were glycoside hydrolase family 16, photosystem I PsaJ, Cytochrome P450 E-class, NDRG|alpha/beta hydrolase fold, and glycosyl transferase family 8 with log2 fold change ranging from −5.4 to −2.6 (Table 2). Interestingly, eight genes were not significant (FDR > 0.05) in the CS-B15sh response to 2-4,D but had a significantly (FDR ≤ 0.05) different responses to 2,4-D compared with the susceptible lines, including nucleotide-diphospho-sugar transferases (Gohir.A06G026500), Myc-type, basic helix–loop–helix (bHLH) domain (Gohir.A13G035300), Ctr copper transporter (Gohir.A03G046900), Cytochrome P450, E-class, group I (Gohir.D06G184100 and Gohir.A12G201300), Heat shock factor binding 1 (Gohir.D11G120200), and two genes of unknown function (Gohir.D12G144700 and Gohir.D06G053801). Four genes, Ctr copper transporter, nucleotide-diphospho-sugar transferases, Myc-type, basic helix–loop–helix (bHLH) domain, and Heat shock factor binding 1, showed an inverted response to 2,4-D in the tolerant compared to the susceptible cotton lines. Interestingly, 16 DEGs were novel cotton transcripts with no functional annotation available (Table 2). Two of these genes, Gohir.A07G188800 and Gohir.D05G048200, were upregulated in response to 2,4-D with eight and threefold change, respectively, while the rest were downregulated in the CS-B15sh response to 2,4-D. Gene co-expression analysis for the 59 DEGs using hierarchical clustering with TM-1 and Pima 379 is presented in Figure 5. GO analysis showed enrichment in genes in pathways involving protein dimerization activity, protein binding, DNA and zinc ion binding, regulation of transcription, hydrolase activity, apoplast, cell wall biogenesis, and organization, as well as photosynthesis (Table 3). Further pathway and network analysis using ptools (Paley et al., 2017) revealed that five genes, including Gohir.A12G201300, Gohir.D08G169900, Gohir.D05G079500, Gohir.A13G049200, and Gohir.D11G154700, are involved in ABA degradation, hormone biosynthesis, L-arginine degradation, and protein modification (Table 4). Both Gohir.A12G201300 and Gohir.D08G169900 are cytochrome P450 with iron ion and heme binding functions and oxidoreductase activity associated with the degradation of ABA into phaseic acid (Figure 6). Gohir.D05G079500 has no known function yet, but is predicted to be involved in ABA biosynthesis (Supplementary Figure 4), while Gohir.A13G049200 and Gohir.D11G154700 are involved in the arginine monooxygenase pathway and protein ubiquitination, respectively (Supplementary Figures 5, 6).

**TABLE 2:** Listing of cotton genes with significantly different responses to 2,4-D in CS-B15sh compared with TM-1 and Pima 379 at 12 h after application of 1.12 kg ae ha^−1^ herbicide^1^.

Cotton gene ID	Description^2^	Response (12 HAT vs. UTD)						Response difference					
		
		Pima 379		CS-B15sh		TM-1		CS-B15shxPima379		CS-B15shxTM-1		TM-1xPima379	
		
		log_2_FC	FDR	log_2_FC	FDR	log_2_FC	FDR	log_2_FC	FDR	log_2_FC	FDR	log_2_FC	FDR
Gohir.A02G076500	–	−0.028	0.983	−5.680	0.000	0.360	0.661	−5.652	0.002	−6.039	0.010	0.387	0.785
Gohir.A03G000035	JmjC/JmjN domain|Zinc finger, C5HC2-type	0.127	0.793	−1.790	0.000	0.678	0.077	−1.917	0.015	−2.468	0.016	0.551	0.362
Gohir.A03G025000	LRR|F-box-like domain superfamily	0.087	0.757	1.284	0.000	0.233	0.308	1.198	0.010	1.052	0.049	0.146	0.702
Gohir.A03G046900	Ctr copper transporter	−0.669	0.008	0.274	0.200	−0.930	0.000	0.944	0.023	1.204	0.023	−0.260	0.429
Gohir.A03G144000	Glycosyl transferase, family 8	−0.641	0.158	−2.599	0.000	−0.743	0.066	−1.958	0.017	−1.857	0.049	−0.102	0.887
Gohir.A05G002900	Glycosyl transferase, family 14	0.049	0.719	−0.778	0.000	−0.254	0.026	−0.827	0.001	−0.523	0.046	−0.303	0.083
Gohir.A05G005600	Glycoside hydrolase family 16	−0.883	0.488	−5.362	0.000	1.427	0.171	−4.479	0.038	−6.789	0.019	2.310	0.166
Gohir.A05G168800	Domain of unknown function DUF1084	−0.359	0.001	−0.721	0.000	−0.271	0.004	−0.362	0.035	−0.449	0.033	0.088	0.534
Gohir.A05G184100	Expansin, cellulose-binding-like domain	1.200	0.043	4.412	0.000	1.746	0.002	3.212	0.004	2.666	0.034	0.546	0.507
Gohir.A05G288000	Protein kinase-like domain superfamily	1.059	0.000	2.225	0.000	1.159	0.000	1.166	0.004	1.067	0.028	0.099	0.763
Gohir.A05G328300	K homology domain, type 1	0.345	0.078	−0.608	0.002	0.351	0.044	−0.953	0.007	−0.959	0.026	0.006	0.987
Gohir.A06G026500	Nucleotide-diphospho-sugar transferases	3.402	0.000	0.721	0.199	3.441	0.000	−2.682	0.019	−2.720	0.040	0.039	0.975
Gohir.A06G049200	–	0.124	0.875	−2.342	0.001	0.911	0.121	−2.466	0.040	−3.253	0.028	0.787	0.410
Gohir.A06G098214	LRR|F-box-like domain superfamily	0.153	0.462	0.947	0.000	0.114	0.528	0.794	0.027	0.833	0.048	−0.039	0.905
Gohir.A07G030300	Homeobox domain	−0.746	0.000	−1.460	0.000	−0.640	0.000	−0.715	0.009	−0.821	0.018	0.106	0.627
Gohir.A07G147600	Zinc finger, FYVE/PHD-type	0.936	0.017	2.402	0.000	0.813	0.019	1.466	0.027	1.589	0.044	−0.123	0.833
Gohir.A07G180900	COBRA, plant	−0.753	0.051	−2.600	0.000	−0.696	0.039	−1.847	0.007	−1.904	0.024	0.057	0.926
Gohir.A07G188800	–	0.902	0.005	2.931	0.000	1.087	0.000	2.029	0.001	1.844	0.010	0.185	0.671
Gohir.A08G151100	Protein ENHANCED DISEASE RESISTANCE 2	0.159	0.467	−0.708	0.001	0.369	0.054	−0.867	0.022	−1.077	0.024	0.210	0.487
Gohir.A08G160000	HAD superfamily	−0.524	0.086	0.687	0.019	−0.587	0.035	1.211	0.025	1.275	0.047	−0.063	0.896
Gohir.A09G112100	RNA recognition motif domain	−1.036	0.005	0.822	0.012	−0.903	0.005	1.858	0.003	1.726	0.023	0.133	0.795
Gohir.A09G213644	Protein kinase domain|NAF domain	−0.111	0.646	0.974	0.000	0.042	0.842	1.085	0.009	0.933	0.049	0.153	0.646
Gohir.A10G079400	Glycosyl transferase, family 1|family 4_5	0.545	0.074	−1.262	0.000	0.162	0.547	−1.807	0.002	−1.424	0.031	−0.383	0.365
Gohir.A10G205200	Zinc finger, GATA-type	−0.089	0.844	−2.417	0.000	−0.004	0.991	−2.327	0.003	−2.412	0.013	0.085	0.895
Gohir.A11G093500	Protein BRANCHLESS TRICHOME-like	0.393	0.008	1.018	0.000	0.389	0.003	0.625	0.012	0.629	0.034	−0.003	0.991
Gohir.A11G127900	Linker histone H1/H5, domain H15	0.205	0.246	0.895	0.000	0.122	0.433	0.690	0.026	0.773	0.038	−0.083	0.755
Gohir.A11G171000	Palmitoyltransferase, DHHC domain	−0.545	0.002	−1.099	0.000	−0.430	0.003	−0.554	0.037	−0.669	0.038	0.116	0.599
Gohir.A11G248400	Myc-type, basic helix-loop-helix (bHLH) domain	−0.390	0.062	0.447	0.023	−0.408	0.031	0.838	0.023	0.855	0.047	−0.018	0.960
Gohir.A11G254800	NDRG|Alpha/Beta hydrolase fold	−0.770	0.024	−2.827	0.000	−1.210	0.000	−2.057	0.001	−1.617	0.028	−0.440	0.341
Gohir.A12G201300	Cytochrome P450, E-class, group I	1.876	0.019	−1.218	0.076	2.819	0.001	−3.094	0.021	−4.037	0.023	0.943	0.388
Gohir.A12G229532	Photosystem I PsaJ, reaction center subunit IX	5.807	0.003	−5.316	0.002	5.926	0.001	−11.122	0.001	−11.242	0.008	0.120	0.963
Gohir.A13G035300	Myc-type, basic helix-loop-helix (bHLH) domain	−2.389	0.001	0.561	0.258	−1.687	0.002	2.950	0.004	2.248	0.047	0.702	0.355
Gohir.A13G049200	Amidase signature (AS) superfamily	0.128	0.438	0.746	0.000	−0.084	0.563	0.618	0.030	0.831	0.023	−0.212	0.349
Gohir.A13G073900	Ran-interacting Mog1 protein	−0.060	0.722	−0.596	0.000	0.284	0.042	−0.536	0.046	−0.880	0.015	0.344	0.109
Gohir.D02G080200	–	−0.158	0.482	−1.473	0.000	−0.509	0.014	−1.315	0.002	−0.964	0.039	−0.351	0.251
Gohir.D02G083700	–	−0.874	0.335	−5.303	0.000	−0.715	0.334	−4.428	0.007	−4.588	0.021	0.160	0.908
Gohir.D03G026100	NRAMP family	0.105	0.661	1.041	0.000	−0.173	0.384	0.936	0.019	1.214	0.019	−0.278	0.377
Gohir.D03G050800	Glycoside hydrolase family 16	−1.024	0.411	−5.348	0.000	−0.728	0.455	−4.324	0.039	−4.620	0.049	0.296	0.870
Gohir.D05G006300	–	−0.402	0.766	−5.281	0.000	2.054	0.042	−4.879	0.021	−7.335	0.010	2.456	0.132
Gohir.D05G017200	–	0.433	0.173	−0.958	0.002	0.500	0.076	−1.391	0.014	−1.459	0.032	0.068	0.893
Gohir.D05G048200	–	0.409	0.067	1.340	0.000	0.382	0.054	0.931	0.018	0.958	0.040	−0.026	0.943
Gohir.D05G079500	–	−0.378	0.094	−1.250	0.000	−0.147	0.464	−0.872	0.029	−1.103	0.027	0.231	0.470
Gohir.D05G153000	–	−0.534	0.053	−2.169	0.000	−1.031	0.000	−1.635	0.002	−1.139	0.045	−0.496	0.189
Gohir.D05G188000	–	0.145	0.383	−0.925	0.000	−0.127	0.381	−1.070	0.001	−0.799	0.027	−0.272	0.231
Gohir.D05G203300	–	−0.082	0.780	−1.291	0.000	−0.169	0.471	−1.209	0.011	−1.122	0.042	−0.088	0.832
Gohir.D06G053801	–	4.758	0.004	−2.283	0.100	6.753	0.000	−7.041	0.009	−9.037	0.013	1.995	0.269
Gohir.D06G102000	–	−0.226	0.790	−4.600	0.000	−1.304	0.051	−4.374	0.003	−3.297	0.039	−1.077	0.305
Gohir.D06G110600	HEAT repeat|Armadillo-type fold	0.101	0.303	−0.296	0.002	0.140	0.103	−0.397	0.021	−0.436	0.035	0.040	0.789
Gohir.D06G184100	Cytochrome P450, E-class, group I	−0.962	0.000	−0.016	0.927	−0.804	0.000	0.946	0.005	0.787	0.042	0.158	0.548
Gohir.D07G023700	Zinc finger, CCCH-type	1.006	0.002	2.208	0.000	0.908	0.002	1.202	0.020	1.300	0.039	−0.098	0.831
Gohir.D07G099500	Eukaryotic translation initiation factor 3 subunit J	−0.483	0.072	−2.386	0.000	−0.704	0.005	−1.904	0.000	−1.682	0.010	−0.222	0.563
Gohir.D08G169900	Cytochrome P450, E-class, group I	−2.056	0.019	−5.076	0.000	−1.525	0.047	−3.020	0.041	−3.551	0.047	0.531	0.663
Gohir.D09G182000	Zinc finger, RING-type	−1.433	0.000	0.626	0.039	−0.750	0.014	2.059	0.001	1.377	0.045	0.682	0.134
Gohir.D11G120200	Heat shock factor binding 1	0.444	0.008	−0.252	0.080	0.457	0.002	−0.696	0.013	−0.709	0.034	0.013	0.961
Gohir.D11G154700	Zinc finger, RING-type	0.023	0.957	1.398	0.000	−0.210	0.529	1.375	0.031	1.608	0.041	−0.233	0.661
Gohir.D11G264500	NDRG|Alpha/Beta hydrolase fold	−0.354	0.233	−2.049	0.000	−0.393	0.132	−1.695	0.002	−1.656	0.016	−0.039	0.936
Gohir.D11G302700	SWEET sugar transporter	0.270	0.478	−1.769	0.000	−0.248	0.448	−2.039	0.003	−1.521	0.047	−0.519	0.314
Gohir.D12G079500	–	−0.011	0.985	−1.548	0.001	0.292	0.440	−1.536	0.039	−1.839	0.041	0.303	0.625
Gohir.D12G144700	–	−3.578	0.000	−0.101	0.870	−3.457	0.000	3.477	0.003	3.356	0.021	0.120	0.904

^1^HAT, hours after treatment; UTD, non-treated controls; FDR, false discovery rate adjusted *p*-value; ^2^No functional annotation available.

**TABLE 3:** Gene ontology (GO) enrichment analysis results for 59 genes responsive to 2,4-D treatment in the herbicide-tolerant cotton line CS-B15sh.

Enriched GO terms	*P*-value	Upland cotton gene(s) within the GO category
Protein dimerization activity (GO0046983, MF)	0.002475248	Gohir.A13G035300.8, Gohir.A13G035300.7, Gohir.A13G035300.6, Gohir.A13G035300.2, Gohir.A11G248400.1
Protein binding (GO0005515, MF)	0.003164557	Gohir.A06G098214.2, Gohir.A06G098214.1, Gohir.A03G025000.8, Gohir.A03G025000.7, Gohir.A03G025000.6, Gohir.A03G025000.5, Gohir.A03G025000.4, Gohir.A03G025000.3, Gohir.A03G025000.2, Gohir.A03G025000.1
Regulation of transcription by RNA polymerase II (GO0006357, BP)	0.007142857	Gohir.A13G035300.8, Gohir.A13G035300.7, Gohir.A13G035300.6, Gohir.A13G035300.2
Hydrolase activity, hydrolyzing *O*-glycosyl compounds (GO0004553, MF)	0.01663586	Gohir.A05G005600.1, Gohir.D03G050800.1
Cell wall (GO0005618, CC)	0.01663586	Gohir.A05G005600.1, Gohir.D03G050800.1
Carbohydrate metabolic process (GO0005975, BP)	0.01663586	Gohir.A05G005600.1, Gohir.D03G050800.1
Cellular glucan metabolic process (GO0006073, BP)	0.01663586	Gohir.A05G005600.1, Gohir.D03G050800.1
Xyloglucan metabolic process (GO0010411, BP)	0.01663586	Gohir.A05G005600.1, Gohir.D03G050800.1
Xyloglucan, xyloglucosyl transferase activity (GO0016762, MF)	0.01663586	Gohir.A05G005600.1, Gohir.D03G050800.1
Cell wall biogenesis (GO0042546, BP)	0.01663586	Gohir.A05G005600.1, Gohir.D03G050800.1
Apoplast (GO0048046, CC)	0.01663586	Gohir.A05G005600.1, Gohir.D03G050800.1
Photosystem I (GO0009522, CC)	0.023076923	Gohir.A12G229532.1
Photosynthesis (GO0015979, BP)	0.023076923	Gohir.A12G229532.1
Zinc ion binding (GO0008270, MF)	0.027874564	Gohir.A10G205200.1, Gohir.A10G205200.2, Gohir.A10G205200.3
Sequence-specific DNA binding (GO0043565, MF)	0.027874564	Gohir.A10G205200.1, Gohir.A10G205200.2, Gohir.A10G205200.3
Positive regulation of transcription, DNA-templated (GO0045893, BP)	0.027874564	Gohir.A10G205200.1, Gohir.A10G205200.2, Gohir.A10G205200.3
Extracellular region (GO0005576, CC)	0.038461538	Gohir.A05G184100.1
Plant-type cell wall organization (GO0009664, BP)	0.038461538	Gohir.A05G184100.1

**TABLE 4:** Ptools pathway analysis results for 59 selected DEGs responsive to 2,4-D treatment in the herbicide-tolerant cotton line CS-B15sh showing the identified pathways and reactions for five DEGs.

DEG	Description	Pathway description	BioCyc pathway ID	Reaction description	BioCyc reaction ID
Gohir.A12G201300.1; Gohir.D08G169900.1	Cytochrome P450, E-class, group I	Abscisic acid degradation to phaseic acid	PWY-5271	2-*cis*-abscisate + a reduced [NADPH-hemoprotein reductase] + oxygen → 8′-hydroxyabscisate + an oxidized [NADPH-hemoprotein reductase] + H_2_O	1.14.13.93-RXN
Gohir.D05G079500.1	Unknown function	Abscisic acid biosynthesis	PWY-695	(+)-*cis*-abscisic aldehyde + H_2_O + oxygen → 2-*cis*-abscisate + hydrogen peroxide + H+	1.2.3.14-RXN
Gohir.A13G049200.1; Gohir.A13G049200.2	Amidase signature (AS) superfamily	L-arginine degradation × (arginine monooxygenase pathway)	ARGDEG-V-PWY	A monocarboxylic acid amide + H_2_O → a monocarboxylate + ammonium	AMIDASE-RXN
				4-guanidinobutyramide + H_2_O → ammonium + 4-guanidinobutanoate	GUANIDINOBUTANA MIDE-NH3-RXN
				2-hydroxyisobutyramide + H_2_O → 2-hydroxy-2-methylpropanoate + ammonium	RXN-17608
				Acetamide + H_2_O → acetate + ammonium	RXN-14728
				Propionamide + H_2_O → propanoate + ammonium	RXN-14727
				Acrylamide + H_2_O → ammonium + acrylate	R311-RXN
				(Indol-3-yl)acetamide + H_2_O → (indol-3-yl)acetate + ammonium	RXNN-404
				Pyrazinamide + H_2_O → ammonium + pyrazine-2-carboxylate	PYRAZIN-RXN
Gohir.D11G154700.1	Zinc finger, RING-type	Protein ubiquitination	PWY-7511	An [E2 ubiquitin-conjugating enzyme]-*S*-ubiquitinyl-L-cysteine + a [protein]-L-lysine → an [E2 ubiquitin-conjugating enzyme]- L-cysteine + a [protein]-N6-monoubiquitinyl-L-lysine + H+	RXN-15561

Of the 59 genes that passed the response comparison filter, 19 had significantly (FDR ≤ 0.05) different expressions between the non-treated samples of the susceptible cotton lines (Supplementary Table 5). Among them were two genes with protein kinase domain (Gohir.A05G288000, Gohir.A09G213644), zinc finger CCCH-type (Gohir.D07G023700), zinc finger RING-type (Gohir.D11G154700, Gohir.D09G182000), JmjC/JmjN domain|zinc finger (Gohir.A03G000035), leucine-rich repeat F-box-like domain (Gohir.A03G025000 and Gohir.A06G098214), amidase signature superfamily (Gohir.A13G049200), NRAMP family (Gohir.D03G026100), protein BRANCHLESS TRICHOME-like (Gohir.A11G093500), HEAT repeat|Armadillo-type fold (Gohir.D06G110600), Cytochrome P450 E-class group 1 (Gohir.D06G184100), homeobox|ELK domain (Gohir.A07G030300), DUF1084 (Gohir.A05G168800), COBRA (Gohir.A07G180900), and three genes of unknown function (Gohir.D12G144700, Gohir.A06G049200, and Gohir.D02G083700).

From the composite DEGs list, some genes were significant (FDR ≤ 0.001) and differentially expressed only in CS-B15sh in response to 2,4-D (treated vs. non-treated) but are not significantly expressed (FDR > 0.001) in TM-1 and Pima 379 plants. These DEGs were not found in the non-treated control pairwise comparisons between CS-B15sh vs. TM-1, CS-B15sh vs. Pima 379, and TM-1 vs. Pima 379. A total of 27 DEGs significantly upregulated in CS-B15sh with 8- to 133-fold change expression were detected (Supplementary Table 6). Functional annotations revealed genes primarily involved in oxidation–reduction and metabolic processes demonstrating that herbicide metabolism is associated with the reduced herbicide injury symptoms observed in CS-B15sh. Several genes involved in transmembrane transporter activities were highly upregulated and explicitly found in CS-B15sh, including Gohir.A05G02220 (SLC26A/SulP transporter domain) and Gohir.A11G129600 (EamA domain|WAT1-related protein) with 133- and 46-fold change, respectively. Other genes involved in plant-type cell wall organizations include the expansin, cellulose-binding-like domain, Gohir.A13G076500, which exhibited 57-fold change of gene expression. Interestingly, several genes involved in oxidation–reduction process (Gohir.D08G249300, Gohir.A06G152100, Gohir.D07G226200, and Gohir.A05G173900) are significant and differentially expressed in CS-B15sh in response to 2,4-D but not in the susceptible lines nor the non-treated control pairwise comparisons.

### Herbicide degradation and metabolism

**FIGURE 7:**
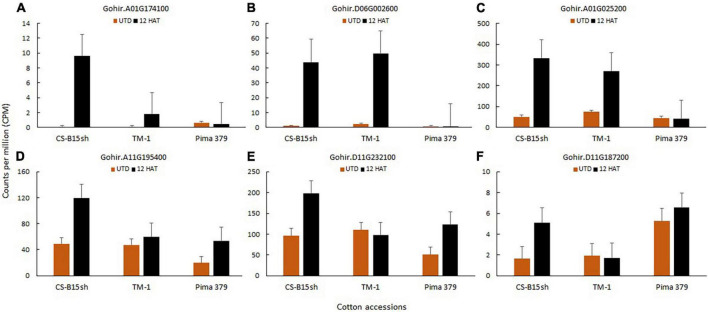
Transcript level expression of DEGs associated with herbicide degradation and metabolism, including flavin monooxygenase, Gohir.A01G174100 **(A)**; FAD-linked oxidase, Gohir.D06G002600 **(B)**; amine oxidase, Gohir.A01G025200 **(C)**; glutathione *S*-transferase, Gohir.A11G195400 and Gohir.D11G232100 **(D,E)**; and Cytochrome P450 monooxygenase, Gohir.D11G187200 **(F)**.

Flavin monooxygenase (FMO) (Gohir.A01G174100) associated with herbicide metabolism is differentially expressed in CS-B15sh. The gene showed 6.8 log_2_ FC, which is twofold higher than TM-1 but is not differentially expressed in Pima 379 (Supplementary Table 7). After 12 h, the transcript level is 5× higher in CSB15sh than TM-1 (Figure 7). Flavin adenine dinucleotide (FAD)-linked oxidase (Gohir.D06G002600) also showed a twofold higher expression in CS-B15sh with 5.7 log_2_ FC compared with the susceptible cotton line TM-1. However, the transcript expression profile showed a similar pattern for CS-B15sh and TM-1 at 43–49 CPM. Other genes associated with herbicide degradation in plants were also highly expressed in CS-B15sh, including amine oxidase (Gohir.A01G025200), glutathione S-transferase (GST) (Gohir.A11G195400 and Gohir.D11G232100), and cytochrome P450 superfamily (Gohir.D11G187200). Log_2_ fold change of amine oxidase is 2.7 and 1.9 for CS-B15sh and TM-1, respectively (Supplementary Table 7). Both GSTs were significantly expressed in CS-B15sh and Pima 379 with 1.1–1.4 log_2_ fold change, while the DEGs were not significant in TM-1. Cytochrome P450 showed 1.6 log_2_ fold change after 2,4-D treatment, while it was not significant in both TM-1 and Pima 379. The transcript levels detected for amine oxidase at 12 h after 2,4-D treatment were 333 and 271 CPM in CS-B15sh and TM-1, respectively. Both DEGs for GST exhibited twofold higher transcript expression in CS-B15sh than TM-1 (Figure 7). Gohir.A11G195400 and Gohir.D11G232100 exhibited 120 and 198 CPM, respectively, in CS-B15sh, while TM-1 exhibited 50% of the CPM detected in both genes. Other genes involved in the metabolism of endogenous substrates including xenobiotics include glycoside hydrolase (Gohir.D09G116300) whose expression was 130-fold higher in treated versus control CS-B15sh plants, which was 6.4 times the change seen in treated versus control TM-1 plants (Supplementary Table 7).

## Discussion

This paper highlights an attempt to understand the interactions of Upland cotton with 2,4-D herbicide at the molecular level. We selected 12 HAT time points for analysis of cotton’s response to 2,4-D based on studies of gene expression responses to abiotic stress in other plants like *Arabidopsis* and *Brassica napus* (Ishitani et al., 1998; Chen et al., 2011). The cotton chromosome substitution line CS-B15sh carries substitution on the short arm of chromosome 15 (D01) from *G. barbadense* L. In previous experiments, a selection of this line (CS-B15sh) showed reduced 2,4-D injury under greenhouse conditions, while TM-1, the genetic background of CS-B15sh, showed sensitivity to 2,4-D and exhibited severe herbicide injury symptoms and plant death. Apparently, Pima 379 is also sensitive to 2,4-D, and thus, the herbicide tolerance in CS-B15sh could be the result of genetic interactions, complementation of alleles of *G. hirsutum* and *G. barbadense* in the development of chromosome substitution line, or formation of transgressive segregants that contributed to phenotypes not observed in the parents such as the level of 2,4-D tolerance observed in CS-B15sh (deVicente and Tanksley, 1993; Rieseberg et al., 1999; Saha et al., 2006). The molecular pathway involved in plant response to 2,4-D has been outlined in previous reports (Grossmann, 2010; Song, 2014; Gaines et al., 2020). Since Upland cotton is sensitive to 2,4-D, it should be interesting to see these molecular key players in the auxin-response pathway differentially expressed with 2,4-D treatment. Several elements of the auxin-signaling pathway outlined in those previous studies were differentially expressed in this study but with varying fold changes observed between the 2,4-D-treated CS-B15sh and TM-1 plants. For instance, the expression of the ubiquitin E3 ligase, part of the ubiquitin-proteasome pathway that degrades AUX/IAA repressor proteins under high auxin/2,4-D conditions, was higher in TM-1 than in CS-B15sh. When AUX/IAAs are degraded, auxin-response factors (ARF) are released, activating auxin-responsive genes and elevating expressions in the herbicide-treated plants (Mockaitis and Estelle, 2008; Guilfoyle, 2015). However, both Gohir.A05G142700 and Gohir.D04G063800 were not differentially expressed in Pima 379 (herbicide 2,4-D-treated vs. untreated) signaling that these genes are not responsive to 2,4-D. The molecular intricacies surrounding how the cotton genome responds to herbicidal 2,4-D and what leads to sensitivity in the plant are still largely unknown. It is possible that other ubiquitin ligase protein families are involved in Pima 379 which is different from those expressed in CS-B15sh and TM-1. Interestingly, three DEGs (Gohir.D08G261100, Gohir.A08G062000, and Gohir.A07G037300) are characterized with PB1 domains showing threefold higher expression in TM-1 compared to CS-B15sh. Proteins with the PB1 domain have been shown to engage in protein–protein interactions with ARF transcription factors (Guilfoyle, 2015) to activate auxin-responsive genes, which leads to severe herbicide injury symptoms as those observed in treated TM-1 plants. This would be consistent with the observation that the expression of an auxin-response factor (Gohir.A01G112300) was threefold higher in treated TM-1 plants than CS-B15sh. In addition, the expression of a gene encoding an F-box (Gohir.A12G196800) is associated with auxin signaling through the promotion of interaction between AUX/IAA proteins and the SCF^TIR1/AFB^ was also higher in TM-1. The SCF^TIR1/AFB^ complex facilitates ubiquitination and subsequent degradation of AUX/IAA repressor proteins, leading to the activation of auxin-responsive genes and disarray of physiological responses caused by 2,4-D at herbicidal rates (Dharmasiri et al., 2005; Song, 2014; Takahashi et al., 2017). The activation and increased expression of hormone-responsive genes such as AP2/ERF proteins and an oxoglutarate/iron-dependent dioxygenase involved in ethylene signaling and biosynthesis indicate that these genes are responsive to auxin herbicide application (Yang and Hoffman, 1984; Houben and Van de Poel, 2019). Future studies in this area are suggested to focus on quantitative gene expression studies of these key elements in the auxin-response pathway to better understand how the auxin/2,4-D response pathway is differentially modulated in CS-B15sh and TM-1 plants exposed to 2,4-D. The effects of auxin herbicides on photosynthesis and ABA metabolism in cotton were similar to recent studies reported (Gaines, 2020; McCauley et al., 2020). Genes associated with photosynthesis were severely downregulated in the 2,4-D-treated cotton plants. This confirms the findings on auxin responses in the weed species *Erigeron canadensis* treated with dicamba and 2,4-D (McCauley et al., 2020). At 12 HAT, genes involved in PS I, PS II, light-harvesting complex, electron transport chain, and chlorophyll biosynthesis were downregulated, indicating the whole scale shut down of photosynthetic process in cotton. Interestingly, both *Gossypium* species (*G. hirsutum* L. and *G. barbadense* L.) showed similar patterns of downregulation of genes involved in photosynthesis, and the differential expression observed is due to genetic background and is not associated with herbicide tolerance in Upland cotton. Although a few genes were found upregulated, the effects of these genes could translate into differences in the coping mechanisms of cotton to 2,4-D and survival from herbicide injury. Similar observations were obtained on ABA and the effects of 2,4-D on cotton, as previously reported in other plants (Gaines, 2020; McCauley et al., 2020). An increase in ethylene and ABA production is well known to be associated with auxin herbicide injury symptoms, such as leaf epinasty, tissue swelling, growth inhibition, tissue decay, and senescence (Grossmann, 2010). Our transcriptomic data also suggested the increased gene expression of DEGs involved in ABA signaling and biosynthesis in Upland cotton treated with herbicidal 2,4-D. Although the ABA biosynthetic gene, 9-*cis*-epoxycarotenoid dioxygenase (NCED), was not detected in the analysis, several genes particularly associated with zeaxanthin synthesis are downregulated. In contrast, genes involved in the synthesis and conversion of intermediates to the formation of ABA from 9′-*cis*-Violaxanthin to xanthoxin and abscisic acid aldehyde exhibited positive log2 fold change. Aspartic peptidase was also reported affecting ABA accumulation in Arabidopsis, while the gene (Gohir.A09G177900) was also found significantly upregulated in all three cotton lines in this study (Kalladan et al., 2017). However, our transcriptome data do not provide evidence on the association of ABA signaling and biosynthesis on herbicide tolerance mechanisms in Upland cotton.

The gene enrichment and pathway analysis of the 59 DEGs responsive to 2,4-D revealed genes associated with photosynthesis and ABA, the five DEGs, including Gohir.A12G201300, Gohir.D08G169900, Gohir.D05G079500, Gohir.A13G049200, and Gohir.D11G154700, present possible pathways that may be involved in herbicide treatment and tolerance response and deserve further investigation. It is interesting to note that Gohir.D05G079500 predicted to be involved in ABA biosynthesis is downregulated in CS-B15sh revealing a relationship with the stress hormone accumulation and possible reduction of herbicide injury symptoms observed in the herbicide-tolerant cotton genotype. While a myriad of molecular patterns and interactions are going on due to 2,4-D’s action, it is worth noting that CS-B15sh exhibits a tolerance level to the herbicide. Comparison of the response of CS-B15sh to those of the two susceptible lines yielded genes involved in cell wall organization and cellular transport mechanisms, indicating possible roles in the diffusion and cell-to-cell movement of 2,4-D active compounds and the tolerance mechanisms exhibited by CS-B15sh to the herbicide. However, some of the genes that showed a significant difference in response to the herbicide were also found significantly expressed in the non-treated plants of TM-1 and Pima 379, indicating that these genes are not associated with defense response to 2,4-D in CS-B15sh. Among these genes are protein kinase-like domain, homeobox domain, zinc finger (CCH-, RING-, and C5HC2-type) protein BRANCHLESS TRICHOME-like, amidase signature (AS) superfamily, NRAMP family, leucine-rich repeat F-box-like domain superfamily, HEAT repeat, COBRA plant, and cytochrome P450 E-class group I (Pace and Brenner, 2001; Roudier et al., 2005; Nevo and Nelson, 2006; Guo et al., 2009). Interestingly, pathway and GO analysis of the DEGs responsive to 2,4-D in CS-B15sh revealed protein dimerization and binding activities, regulation of transcription, hydrolase activity, cell wall, and carbohydrate metabolic processes are among the most enriched pathway signaling regulation of proteins such as enzymes, co-factors, ion channels, and transcription factors (Marianayagam et al., 2004). Both protein containing assembly and hydrolase activity were also associated with the non-target site resistance phenotypes to herbicide phenylurea chlorotoluron and aryloxyphenoxypropionate fenoxaprop acid in black-grass weeds (Franco-Ortega et al., 2021). Glycoside hydrolase is primarily involved in the processing of carbohydrates and works on the metabolism of endogenous substrates, including xenobiotics (Oesch-Bartlomowicz and Oesch, 2007). Other genes involved in xenobiotic metabolism like glycosyltransferases were expressed only in CS-B15sh (Brazier-Hicks et al., 2007). The downregulation of these genes after 2,4-D treatment signals a different molecular pathways contributing to the herbicide tolerance exhibited by CS-B15sh. The three cotton lines we used exhibited a differential response to 2,4-D, and CS-B15sh showed some degree of tolerance to the herbicide compared to TM-1 and Pima 379. Likely, some physiological and metabolic processing of the herbicide compound within the plant occurs with the high transcript expression patterns of DEGs associated with herbicide metabolism, such as those involved in oxidation–reduction processes, including GSTs, amine oxidase, and FAD-linked oxidase. Although recent reports have implicated L-lectin domain-containing receptor kinases, ABC transporters, and cytochrome P-450s in the tolerance of various weeds to 2,4-D (Giacomini et al., 2020; Goggin et al., 2020). We found examples of these genes upregulated in 2,4-D-treated CS-B15sh and TM-1 plants. However, there was no significant increase in the expression of any of these genes that will contribute to herbicide tolerance in our study. Future experiments should focus on elucidating the genetic functions of DEGs highly expressed in CS-B15sh, such as glutathione S-transferase and flavin monooxygenase, because these genes are involved in herbicide detoxification. It would be interesting to do further assays to validate its functions in terms of herbicide tolerance in cotton, develop genetic markers useable as molecular markers for 2,4-D tolerance in marker-assisted breeding, and therefore contribute to the genetic improvement of modern Upland cotton varieties.

## Conclusion

In summary, the interaction of cotton to 2,4-D herbicide at the molecular level was elucidated in this study. Several genes involved in the auxin (2,4-D) response pathway previously reported have been detected. Downregulation of a whole suite of genes involved in photosynthesis was observed. The response of the 2,4-D-tolerant line CS-B15sh showed upregulation of genes involved in the oxidation–reduction process. It will be interesting to conduct a follow-up investigation on the specific function of these genes in cotton’s response to 2,4-D. This information will allow the design of appropriate breeding strategy toward the development of modern Upland cotton varieties with improved tolerance to herbicides.

## Data availability statement

The datasets presented in this study can be found in online repositories. The names of the repository/repositories and accession number(s) can be found below: https://www.ncbi.nlm.nih.gov/bioproject/PRJNA824818.

## Author contributions

TT, LP, ZM, JD, and DP designed the experiments. LP conducted the experiments and wrote the original version of this manuscript. RM and MA performed data analysis. All authors have read, contributed, and approved the manuscript.

## Footnotes

1
http://www.bioconductor.org

2
http://ptools.cottongen.org/

3
https://bioconductor.org/packages/release/bioc/html/fgsea.html

## Supplementary material

Supplementary Figure 1 MapMan analysis showing the overview of metabolic pathways in TM-1. Each data point (square) represents a DEG gene. Blue and red colors indicate the DEGs are upregulated and downregulated, respectively.

Supplementary Figure 2 MapMan analysis showing the overview of metabolic pathways in Pima 379. Each data point (square) represents a DEG gene. Blue and red colors indicate the DEGs are upregulated and downregulated, respectively.

Supplementary Figure 3 Gene co-expression heatmap with hierarchical clustering of DEGs associated with photosynthesis.

Supplementary Figure 4 Ptools pathway analysis on hormone biosynthesis (Gohir.D05G079500).

Supplementary Figure 5 Ptools pathway analysis on L-arg degradation (Gohir.A13G049200).

Supplementary Figure 6 Ptools pathway analysis on protein modification (Gohir.D11G154700).

Supplementary Table 1 List of DEGs in the composite set (union of 3 genotypes).

Supplementary Table 2 Gene ontology.

Supplementary Table 3 List of DEGs associated with photosynthesis.

Supplementary Table 4 List of DEGs associated with ABA signaling and biosynthesis.

Supplementary Table 5 Gene expression in UTD (untreated) and 12 HAT contrasts of the 59 DEGs.

Supplementary Table 6 List of DEGs unique and significantly expressed (FDR < 0.0001) in CS-B15sh (treated vs. untreated).

Supplementary Table 7 DEGs associated with herbicide metabolism.

Supplementary Table 8 Read counts and mapping rate.
