# Supplementary figures and images for: Transcriptome analysis of the 2,4-dichlorophenoxyacetic acid (2,4-D)-tolerant cotton chromosome substitution line CS-B15sh and its susceptible parental lines G. hirsutum L. cv. Texas Marker-1 and G. barbadense L. cv. Pima 379
Source: Front Plant Sci. 2022 Aug 22;13:910369. doi: 10.3389/fpls.2022.910369 (PMC9441920; doi:10.3389/fpls.2022.910369)

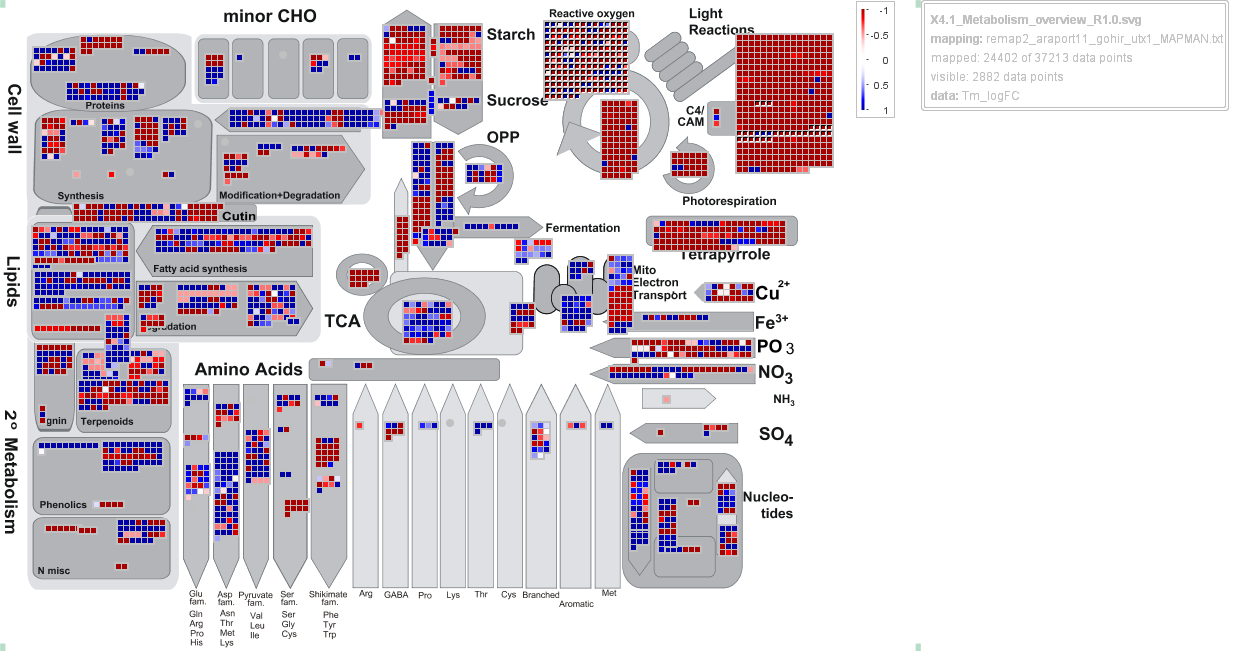

Supplement: Supplementary Figure 1 — MapMan analysis showing the overview of metabolic pathways in TM-1. Each data point (square) represents a DEG gene. Blue and red colors indicate the DEGs are upregulated and downregulated, respectively. [file Image_1.JPEG]

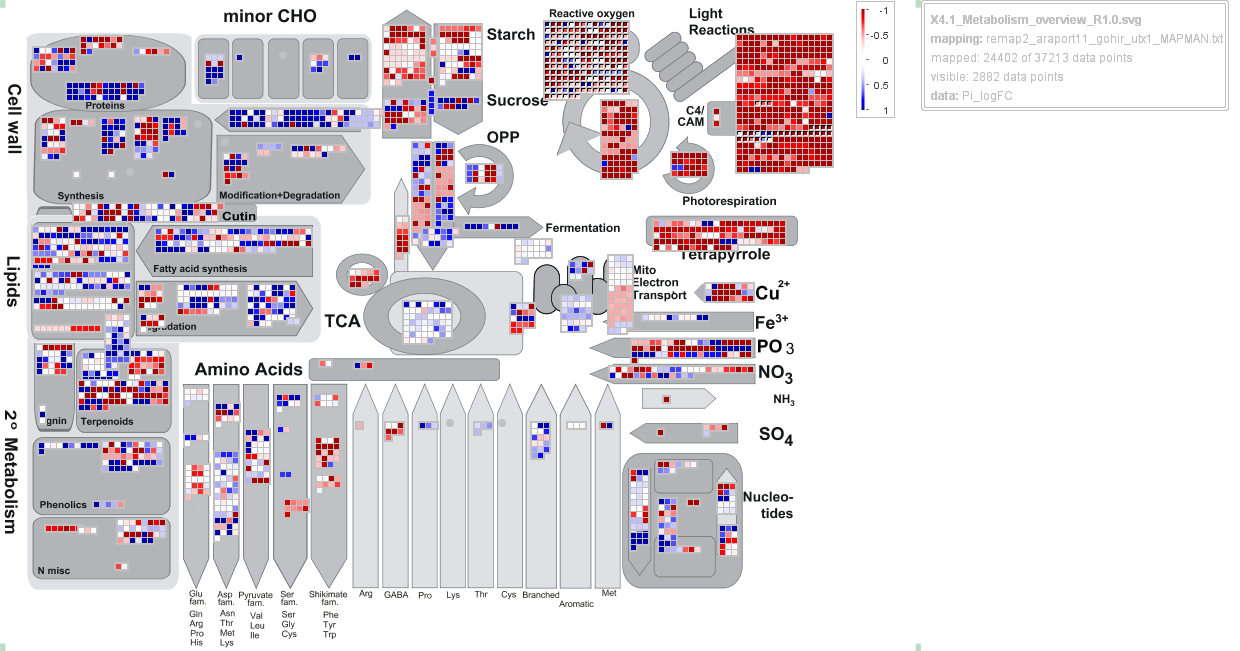

Supplement: Supplementary Figure 2 — MapMan analysis showing the overview of metabolic pathways in Pima 379. Each data point (square) represents a DEG gene. Blue and red colors indicate the DEGs are upregulated and downregulated, respectively. [file Image_2.JPEG]

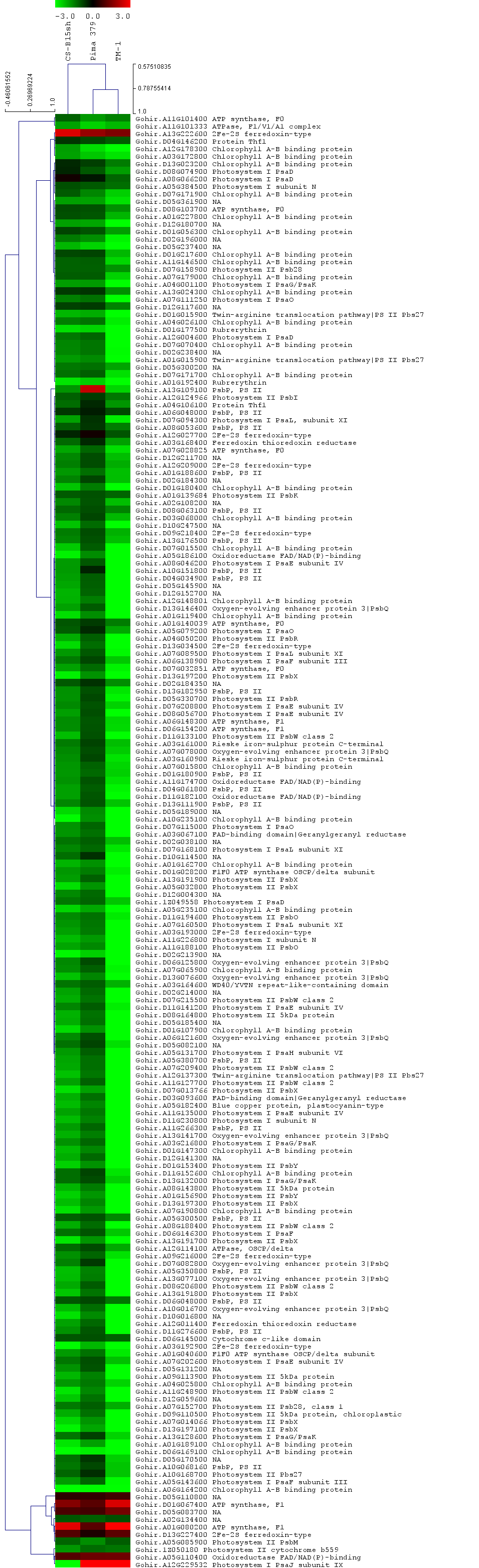

Supplement: Supplementary Figure 3 — Gene co-expression heatmap with hierarchical clustering of DEGs associated with photosynthesis. [file Image_3.JPEG]

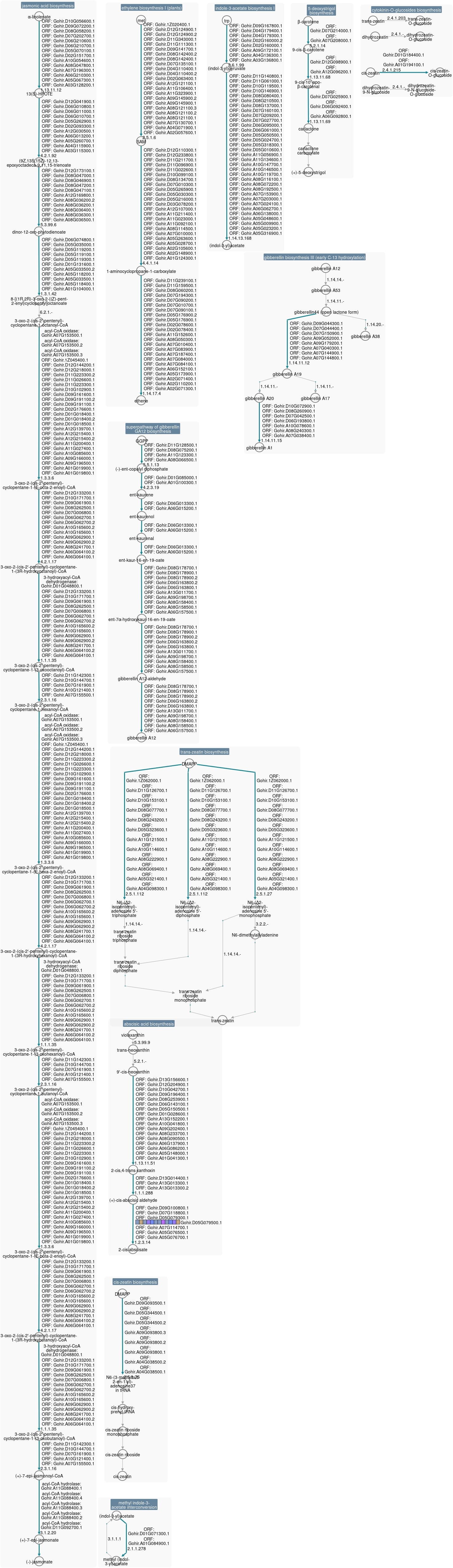

Supplement: Supplementary Figure 4 — Ptools pathway analysis on hormone biosynthesis (Gohir.D05G079500). [file Image_4.JPEG]

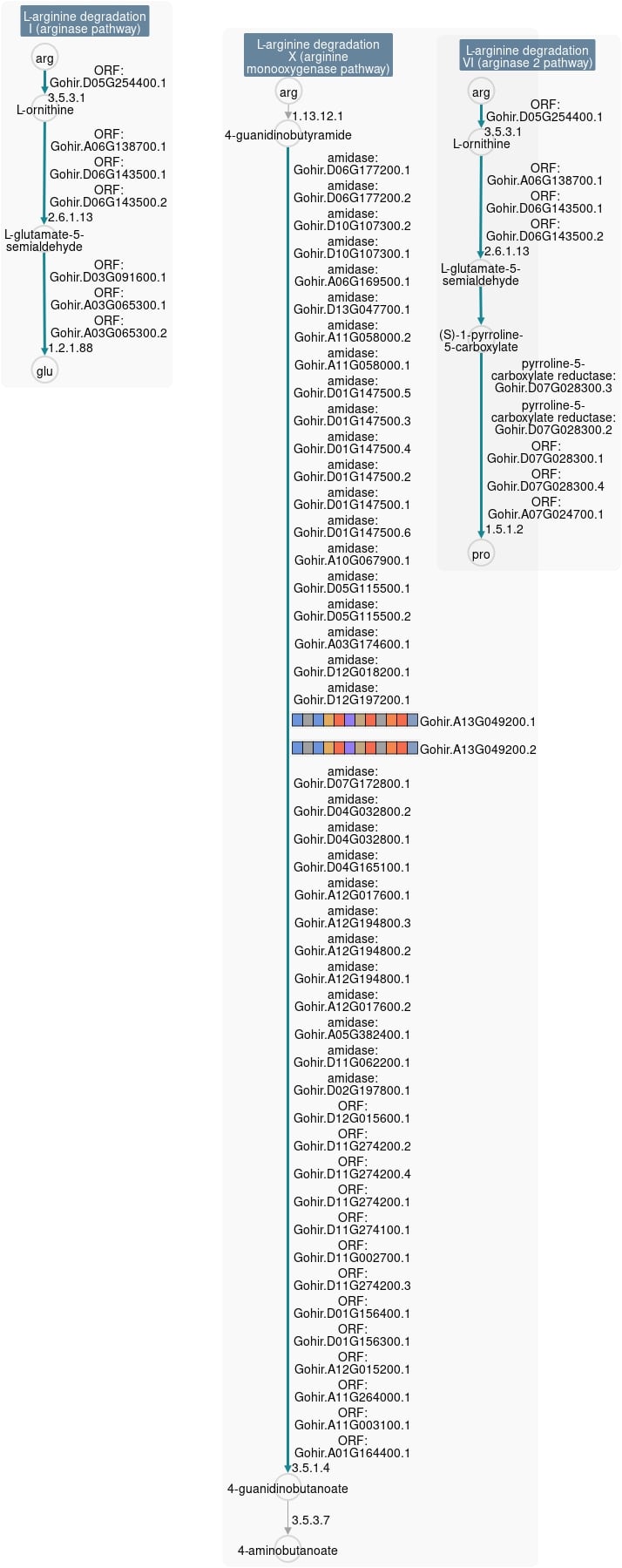

Supplement: Supplementary Figure 5 — Ptools pathway analysis on L-arg degradation (Gohir.A13G049200). [file Image_5.JPEG]
